# A Comparative Clinical Study of a Novel Pre-colonoscopy Bowel Capsule Preparation Against Two Commercially Available Liquid Preparations

**DOI:** 10.3389/fmedt.2020.622252

**Published:** 2021-02-09

**Authors:** Harriet Kingston-Smith, Anoja W. Gunaratne, John Saxon, Sanjay Ramrakha, Marie Vic M. Dawson, Annabel Clancy, Antony Wettstein, Thomas J. Borody

**Affiliations:** Centre for Digestive Diseases, Sydney, NSW, Australia

**Keywords:** cathartics, colonoscopy, colonic polyps, polyethylene glycol, capsule bowel preparation

## Abstract

**Background and Aims:** Colonoscopy surveillance depends on effective bowel preparation. Inadequate bowel preparation can lead to inaccurate clinical diagnosis, insufficient visualization of the colon and increased risk of missed diagnosis. This study aimed to compare the efficacy and safety of a novel Capsule Bowel Preparation (RitePrep), high-volume (2L) polyethylene glycol electrolyte solution (MoviPrep®) and low-volume (1L) polyethylene glycol electrolyte solution (Plenvu™).

**Methods:** Patients (*n* = 120) were divided into three groups and were administered either RitePrep, MoviPrep® or Plenvu™ as a pre-colonoscopy bowel preparation followed by a colonoscopy at a single center. Validated Boston Bowel Preparation Score (BBPS) and bubble score were used to evaluate bowel cleanliness. Blood tests were also evaluated. The scores and the blood results were analyzed using Kruskal-Wallis and Chi-squared tests.

**Results:** A total of 120 patients (median age of 55; 57 males) [RitePrep (*n* = 40), MoviPrep® (*n* = 40) and Plenvu™ (*n* = 40)] were included in the study. RitePrep was the most effective method for cleansing the bowel, with a significantly higher median BBPS compared to MoviPrep® and Plenvu™ (*p* = 0.006 and 0.024, respectively). Nearly 50% of the patients in Plenvu™ group showed increased serum osmolality disturbance. Nausea and vomiting were higher in Plenvu™ and MoviPrep® groups than RitePrep group.

**Conclusions:** RitePrep was demonstrated to be a more effective and safe preparation than the other two preparations. RitePrep was not only well-tolerated by all patients; the preparation sufficiently cleared the ascending, transverse, and descending colon, enabling optimal visualization for the clinician. RitePrep was also much safer than the comparators, with no alteration in electrolytes measured. For both the clinician and the patient, RitePrep was the preferred preparation.

## Introduction

The effectiveness of a colonoscopy procedure relies on an adequately prepared bowel. Bowel preparation aims to completely evacuate and clean the bowel before conducting a colonoscopy procedure, which allows the doctor to properly examine the colon. Poorly prepped bowels can lead to impaired visibility during the examination, increased potential for missed and potentially cancerous lesions, prolonged procedure duration, and repeat procedures (1). Poor bowel preparation additionally leads to impaired detection rate of polyps (2). There is also an increased risk of bowel perforation, which can be caused from blinded maneuvers into fecally obscured diverticulae (3).

A variety of different bowel preparations are available to be used to clean the bowel. However, effective bowel preparations have yet to achieve acceptance by both the clinician and the patient. Many patients cannot tolerate the currently available bowel preparations which often sacrifice palatability and can cause severe electrolyte disturbances (4). Tolerability is a major factor in good bowel preparations, with the poor palatability of bowel preparation often leading to patients' trepidation in undergoing the screening colonoscopy procedure (1).

Problems in the bowel preparation phase can result from the physiological disturbance of fluid and electrolyte shifts, which stem from the purgative effect. Side effects can occur as a result of clinically significant hyponatremia and include confusion, headaches, seizures and comas (5).

Currently marketed bowel preparations such as Plenvu™, MoviPrep®, ColonLYTELY®, and GoLYTELY® contain Polyethylene Glycol (PEG) which is considered advantageous as it generally results in lower electrolyte shifts. However, PEG solutions still have significant compliance problems because of palatability and volume issues to reach adequate bowel preparation (6). Newer PEG products have gradually reduced volume and become more concentrated with splitting dose for better cleanliness (7). However, the early safety features associated with first generation PEG product may no longer apply to the new family of PEG products (8).

To improve on the poorly-tolerated large volumes of solution has been the encapsulation of active ingredients. Sodium phosphate tablets showed improved palatability and patient acceptance and were equally or more effective than PEG products (9). Unfortunately, reported renal damage in some patients after taking sodium phosphate tablets has significantly limited their use and are highly unsuitable for people with chronic kidney disease, diabetes and the elderly population (9).

Results from randomized controlled trials have shown that 25% of bowel preparations are sub optimal (10, 11). The ideal bowel preparation should clean the colon of all fecal matter without altering the colonic mucosa. The preparation should be well-tolerated by the patient, palatable, and not cause any fluid shifts and electrolyte imbalance.

At the Center for Digestive Diseases, we developed a novel Capsule Bowel Preparation (RitePrep). RitePrep incorporates the stimulant laxative, sodium bisoxatin with the addition of electrolytes, packaged in a capsule. We compared the safety and efficacy profile of RitePrep and compared to two currently marketed PEG-based preparations: High volume (2 liters) polyethylene glycol electrolyte solution (MoviPrep®) and Low volume (1 liter) polyethylene glycol electrolyte solution (Plenvu™). We examined the polyp detection rates (adenoma /sessile serrated adenoma), time to complete colonoscopy and tolerability of RitePrep, MoviPrep® and Plenvu™. Safety was also assessed by examining the levels of sugar, electrolyte and osmolality in blood samples.

## Materials and Methods

### Study Design

This was a single-center, prospective pilot study of outpatients undergoing bowel preparation and colonoscopy from July 2018 to November 2018. Inclusion criteria included patients undergoing colonoscopy procedure who: required bowel preparation, were between 18 and 85 years of age, and consented in writing. Patients were excluded if they had renal impairment or disease, uncontrolled diabetes mellitus, were taking contraindicated medications or medications which may change electrolyte balance or did not require bowel preparation for their colonoscopy.

At the time of this study, three different bowel preparation methods were recommended for patients undergoing a colonoscopy at our center: (1) MoviPrep® (Salix Pharmaceuticals, USA); (2) Plenvu™ (Salix Pharmaceuticals, USA) and (3) RitePrep. Patients were recommended one of these three preparation methods at the discretion of their clinician, taking into account their current medications, medical history, and reaction to any previous bowel preparation products (Table 1). All patients who were recruited to the study were provided an instruction sheet to avoid fiber-rich food for 3 days prior to the procedure. On the day before the procedure, clear fluid diets were recommended. Clear fluids were specified as water, black tea, black coffee with no milk or sugar, unsweetened 100% fruit juice, clear soups or broths, stock cubes in water, artificially sweetened jelly (no red or green colored jelly) and fizzy drinks without sugar.

**Table 1:** Bowel preparation composition and inclusion/exclusion criteria.

**Bowel preparation**	**Inclusion criteria**	**Exclusion criteria**	**Composition**	**Volume/dose**
Capsule bowel preparation (RitePrep)	Patients undergoing colonoscopy and between 18–85 years of age	Renal impairment or disease Liver impairment or disease Cardiac Disease Medications that affect blood electrolyte levels, including; ACE inhibitors Selective Serotonin Reuptake Inhibitors Pregnant or lactating	Bisoxatin, Erithritol, Magnesium Sulfate, Sodium Sulfate and Potassium Sulfate	43 capsules Bottle A = 18 capsules Bottle B = 10 capsules Bottle C = 15 capsules (capsule consumption schedule see Appendix A in Supplementary Material)
Plenvu™ (L-PEG)	Patients undergoing colonoscopy and between 18 and 85 years of age	Known or suspected: gastrointestinal obstruction or perforation Ileus Disorders of gastric emptying Phenylketonuria G6PD Deficiency Toxic Megacolon Hypersensitivity to active ingredients	PEG, sodium sulfate, sodium chloride, potassium chloride, ascorbic acid and sodium ascorbate	3 sachets−1L(see instruction to prepare and consume the powder in pdf^a^)
MoviPrep® (H-PEG)	Patients undergoing colonoscopy and between 18 and 85 years of age	Hypersensitivity to active ingredients	PEG, macrogol, sodium sulfate anhydrous, sodium chloride, potassium chloride, ascorbic acid and sodium ascorbate	4 sachets−2L(see instruction to prepare and consume the powder in pdf^b^)

a *https://www.norgine.de/wp-content/uploads/2018/03/180607_02_Einnahmeanleitung_A4_eng.pdf*.

b *https://centrefordigestivediseases.com/wp-content/uploads/2018/07/English-amended.pdf*.

Patients who were in the RitePrep group received 43 capsules. The capsules were manufactured in an Australian registered pharmacy according to a protocol and standard operation procedures. The 43 capsules were divided into three bottles containing 18, 15, and 10 capsules respectively. On the day preceding to their procedure, the patients were instructed to take 6 capsules with one glass (250 ml) of water, every half an hour from 3 p.m. If 15 bowel motions were passed, patients were asked to stop taking the capsules but could continue to take clear fluids until 12 am. If they opened their bowel within 2 h but had not passed more than 15 bowel motions, then the patients were asked to take the capsules from the second bottle, with five capsules with one glass of water every half an hour from 7 p.m. If patients did not open their bowel by 5 p.m., the patients were advised to take five capsules from both the second and third bottle with water, every half an hour (see the Appendix A in Supplementary Material).

Patients in the Plenvu™ and MoviPrep® groups received information and instructions to complete the bowel preparations as per the manufacturer's standards (web PDFs including Norgine 2018 and MOVIPREP Outpatient 2018) (12, 13). In brief, patients who were in the Plenvu™ group were asked to consume a maximum 1 L of the Plenvu™ solution and patients in the MoviPrep® group were asked to consume 2 L MoviPrep® solution. Patients consumed their solutions in two separate doses according to their procedure time. One dose included either 500 ml Plenvu™ solution or 1 L MoviPrep® solution that was consumed within 90 min. They were also asked to consume 500 ml of clear fluids during these 90 min.

Colonoscopy procedures were conducted using an EVIS EXERA III Xenon (CLV-190) high-definition colonoscope by qualified two gastroenterologists with more than 20 years' experience. A sedationist was present to assess and observe patients' vital signs during the entire procedure. The time was recorded at the beginning and withdrawing the colonoscope. Immediately after completing the colonoscopy, the gastroenterologist and sedationist independently rated the adequacy of the bowel preparations using the validated Boston Bowel Preparation Score (BBPS) score (14). The BBPS is a 9-point scale assessing the ascending (3-point), transverse (3-point), and descending colon (3-point), where 0 = inadequate (unprepared colon segment with mucosa unable to be visualized because of solid stool that cannot be cleared); and 3 = excellent (entire mucosa of colon segment can be visualized with no residual staining, small fragments of stool or opaque liquid) (14). Polyp detection rate (PDR) was also recorded by the gastroenterologist by reporting polyp numbers in the ascending, transverse, and descending colon. Intraluminal gas bubbles were assessed in the colon and graded as follows: A = absent/ minimal bubbles; B = moderate bubbles; and C = numerous bubbles (15).

From each patient, a blood sample was collected to measure sodium, potassium, magnesium, creatinine, urea/nitrogen and serum osmolality at the time of colonoscopy.

Patients also completed a questionnaire regarding tolerability, taste, compliance, satisfaction and willingness to repeat the same preparation for future procedures. Patients were also asked to report on symptoms associated with the bowel preparation including nausea, vomiting, abdominal pain, and number of bowel motions. Patients also completed a baseline gastrointestinal symptom questionnaire and a drinking log.

Patient characteristics including demographic data, medical history, concurrent medications, and current symptoms were tabulated from patients' medical records.

#### Statistics

Statistical analysis using descriptive statistics were performed using GraphPad Prism version 8 for Windows, GraphPad Software, (La Jolla California USA, www.graphpad.com). The proportion of patients with demographic data were summarized as numbers and percentages between the three groups. Inter-rater reliability of the attending gastroenterologist and sedationists was assessed using Cohen's kappa statistic to compare agreement for each BBPS score. A Kruskal-Wallis Test was used to compare the differences in median BBPS scores between the three preparation groups. *Post-hoc* analysis with Mann Whitney U tests were used for pair wise comparisons between groups. The relationship between bubble scores, PDR and laboratory test results and the preparation type was assessed using Chi-Squared analysis. Statistical significance was set at 5%.

#### Study Outcomes

The primary objective of this pilot study was to demonstrate that RitePrep capsules provided an “adequate” bowel preparation (measured by BBPS ≥ 6) when compared to either MoviPrep® or Plenvu™. The secondary outcome of this pilot study was to show that RitePrep had no or limited effect upon osmolarity.

## Results

A total of 120 patients (57 M, average age: 55 years) (40 RitePrep, 40 MoviPrep®, 40 Plenvu™) were included in the study (Table 2). Patients were distributed randomly to each of the 3 groups. No statistical differences were observed between the 3 testing groups, however there were more patients with Crohn's Disease distributed to the RitePrep group (Table 2).

**Table 2:** Demographics of the participants (*n* = 120).

**Description**	**RitePrep (*N* = 40)**	**Plenvu^**TM**^ (*N* = 40)**	**MoviPrep^®^ (*N* = 40)**	***P*-value**
Males *n* (%)	16 (40)	22 (55)	19 (47.5)	0.66
Age median (SD)	56.5 (17.9)	57.5 (14.4)	56 (16.1)	0.41
Time for the procedure in	30 (11.1)	26.5 (8.1)	25 (9.1)	0.94
minutes median (SD)				
Constipation *n*	3	2	3	0.88
Crohn's Disease *n*	8	2	1	**0.02**
Ulcerative Colitis *n*	2	3	0	0.23
Bowel cancer Screening *n*	15	23	20	0.28
Other Indications^*^n	12	10	16	0.34

* *Symptoms under investigation*.

*Bold values mean significant*.

The inter-rater reliability between the gastroenterologist and the sedationist BBPS was assessed using Cohen's kappa coefficient (Table 3). During the course of this study, 120 patients from the three different arms were scored. From this, the number of observed agreements was 94 (78.3%). The number of agreements expected by chance: 61.0 (50.83% of the observations), providing a Weighted Kappa = 0.610. Assessed this way, the strength of agreement is considered to be “good,” and so only the gastroenterologists scores were used for further analysis.

**Table 3:** Assessment of Inter-Rater Reliability between gastroenterologist and sadationist BBPS.

**BBPS scores**		**Gastroenterologist**			
		**Poor**	**Moderate**	**Excellent**	**Total**
**Sedationist**	Poor	**3**	2	0	5
	Moderate	4	**24**	12	40
	Excellent	0	8	**67**	75
	Total	7	34	79	120

*Bolded numbers shows agreement between both assessors*.

**Figure 1:**
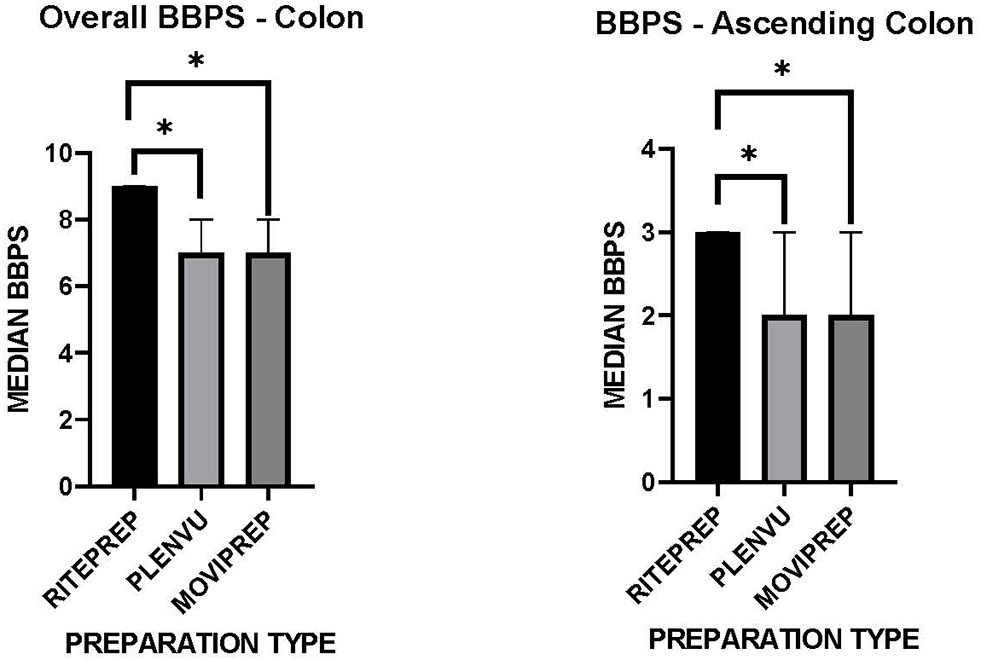
Doctor's BBPS evaluations for three preparation types. *Significant <0.05.

Adequate bowel preparation was defined as a BBPS score of ≥ 6, whereas inadequate bowel preparation was defined as a BBPS score of <6. All preparations resulted in an “adequate” bowel preparation, scoring ≥6. However, the median BBPS score for patients receiving RitePrep was 9 (IQR 3) compared to 7 (IQR 3) and 7 (IQR 2) for Plenvu™ and MoviPrep® (Figure 1, *p* = 0.008 and 0.007 respectively). Further analysis, focusing on the ascending colon was also conducted. BBPS scores were significantly different in the more difficult to view ascending colon between the three preparation groups. *Post-hoc* analysis revealed a statistically higher BBPS score for RitePrep (3) compared to Plenvu™ (2) and MoviPrep® (2) (Figure 1, *p* = 0.006 and 0.024, respectively), but not between Plenvu™ and MoviPrep® (*p* = 0.50).

Eighty percent of patients treated with RitePrep had no bubbles in their colon, compared to 67.5 and 52.5% treated with Plenvu™ and MoviPrep® respectively (Table 4). Nearly 10% of patients had severe bubbles in the Plenvu™ and MoviPrep® groups. In contrast, none of the patients treated with RitePrep recorded severe bubbles (Table 4). PDR was analyzed only for the bowel screening patients and no differences were seen between the groups (Table 4).

**Table 4:** Differences in Bubble scores and Polyp Detection Rates between bowel preparation groups.

	**RitePrep**	**Plenvu^**TM**^**	**MoviPrep®**	***P*-value**
**Bubble scores**				
None *n* (%)	32 (80)	27 (67.5)	21 (52.5)	0.08
Moderate *n* (%)	8 (20)	10 (25)	15 (37.5)	
Severe *n* (%)	0	3 (7.5)	4 (10)	
Polyp detection rates^*^				
Bowel cancer Screening patients *n*	15	23	20	0.53
None *n* (%)	10 (66.6)	19 (82.6)	15 (75.0)	
Polyp detected *n* (%)	5 (33.3)	4 (17.4)	5 (25.0)	

* *Screening/surveillance patients only*.

The association between abnormal laboratory tests (above or below the standard reference range) and bowel preparation types were examined (Table 5). There was a statistically significant association between elevated serum osmolality and Plenvu™, with 18 patients (45%) showing an increase in serum osmolality outside the reference range, compared to RitePrep (*n* = 3) and MoviPrep® (*n* = 2) (Table 5, *p* < 0.001). Due to the small number of results detected outside of the reference range for sodium, potassium, magnesium, bicarbonate, phosphate, and albumin, further analysis was unable to be completed.

**Table 5:** Bowel preparation type and effect on serum osmolality and electrolytes.

	**Results**	**Preparation type n**			***P*-value**
		**RitePrep**	**Plenvu^**TM**^**	**MoviPrep^®^**	
Serum	H	3	18	2	**<0.001**
osmolality	N	30	21	37	
	L	0	0	0	
Glucose	H	1	3	1	0.095
	N	20	18	30	
	L	3	7	4	
Sodium	H	1	6	1	N/A
	N	38	34	38	
	L	0	0	1	
Potassium	H	1	0	0	N/A
	N	34	37	35	
	L	1	2	1	
Bicarbonate	H	0	30	5	N/A
	N	0	5	1	
	L	0	0	5	
Calcium	H	0	1	0	N/A
	N	37	39	40	
	L	3	0	0	
Phosphate	H	5	8	1	N/A
	N	34	32	37	
	L	0	0	1	
Albumin	H	3	1	1	N/A
	N	35	38	39	
	L	2	0	0	

*H, High (above reference range); N, Normal; L, Low (below reference range); N/A, No further analysis able to be performed*.

*Bold values mean significant*.

Patients' satisfaction and self-reported symptoms with the bowel preparation were reported 47.5% (*n* = 19) in RitePrep, 62.5% (*n* = 25) in Plenvu™ and 82.5% (*n* = 33) in MoviPrep® groups (Table 6). Patients reported abdominal cramping, headaches, nausea, vomiting and urgency to pass a motion as the most common side effects after taking a bowel preparation solution. Interestingly, patients taking MoviPrep® reported significantly less bowel motions than those taking the RitePrep or Plenvu™ (Table 6, *p* = 0.02). A significantly higher number of patients experienced abdominal pain in the Plenvu™ group (*p* = 0.008). Nearly a half of the Plenvu™ patients (48%) had nausea and vomiting compared to the patients who took RitePrep (21%) and MoviPrep® (24%) (Table 6).

**Table 6:** Patients' satisfaction and self-reported symptoms with the bowel preparation.

**After having the bowel preparation, patient reported;**	**RitePrep (*N* = 19)**	**Plenvu^**TM**^ (*N* = 25)**	**MoviPrep^®^ (*N* = 33)**	***P*-value**
Bowel motions median (SD)	13.5 (5.46)	13 (5.62)	10 (4.85)	**0.02**
Effective bowel cleaning *n* (%)	18 (95)	25 (100)	32 (97)	0.20
Nausea *n* (%)	4 (21)	10 (40)	5 (15)	0.10
Vomiting *n* (%)	0	2 (8)	3 (9)	0.42
Abdominal pain *n* (%)	2 (11)	5 (20)	2 (6)	**0.008**
Patients' willingness to take same preparation *n* (%)	19 (100)	16 (64)	27 (82)	**0.007**

*Bold values mean significant*.

## Discussion

Visualization of the colon is critical to the gastroenterologist to adequately diagnose abnormalities in the bowel. Indeed, equally important to the patient is a bowel preparation that is well-tolerated, palatable and with minimal side-effects.

Evidence suggests that one in four patients do not have adequate bowel cleanliness for colonoscopy (16). Adequate preparation of the ascending colon is commonly worse than the descending colon (4, 17). Visualization of the ascending colon is challenging (18), and has shown to have a positive correlation with the PDR (19, 20). RitePrep is encapsulated, safe to the patient and demonstrates excellent visualization of the colon, in particular the ascending colon. Using the established BBPS system, RitePrep demonstrated significantly superior visualization of the ascending, transverse, and descending colon compared to the widely used Plenvu™ and MoviPrep® solutions.

The efficacy of RitePrep is due to the active ingredient Bisoxatin. Bisoxatin is stimulant laxative which increases peristalsis and inhibits the absorption of water and ions in the intestine (FAMPH: Bisoxatin Summary of Product Characteristics). Bisoxatin has been used to effectively treat chronic constipation and is known to be well-tolerated with negligible side effects and free from toxic effects (21). When compared with bisacodyl for treatment of functional constipation, Bisoxatin has been reported to have superior clinical results and reduced side effects (22).

All patients who had RitePrep were willing to have same preparation again (100%). This may reflect the patients' preference for the capsule form including palatability and convenience. RitePrep does not have any taste and the patients can tailor their treatment by stopping once they have experienced 15 bowel motions. Similarly, in a study of 845 patients (*n* = 420 tablet and *n* = 425 PEG solution groups), showed greater compliance with the tablet (94%) compared to the PEG solution (57%) (*p* < 0.0001). This study also found the tablets were easier to take with 88% rating them as “easy” compared to 61% of patients taking the PEG solution (23). Another randomized controlled trial comparing bowel preparation tablets vs. a 2 liter PEG solution for bowel preparation in 411 patients showed a superior tolerance with the tablets (77 vs. 42%) (24). Overall, encapsulated bowel preparation appears to increase acceptance and palatability for patients.

Another advantage of RitePrep was its favorable side effect profile. When we assessed the blood profiles of the patients enrolled in this study, 45% of patients receiving the low volume PEG solution, Plenvu™ demonstrated dangerous osmolarity levels. Previous trials of Plenvu™ have reported hypernatremia and clinical dehydration in patients (25). This is of concern for patients outside the trial setting who may be less healthy and/or elderly. According to a recent editorial, the manufacturers of Plenvu™ now recommends in their packaging insert for the patient to drink an addition 2 liters of clear fluid when using Plenvu™ (25). This clearly defeats the purpose of low volume PEG preparations. Additionally, in our Plenvu™ group, nausea was prevalent and when surveyed, 36% patients would not take this preparation again for future colonoscopies, due to the severity of side effects.

We acknowledge there were limitations to the current study. Firstly, the study was a pilot hence no power calculation was provided. Secondly, patients were not randomized to the three study arms using a non-biased computerized system or randomization system. Selection bias cannot be ruled out. And finally, all patients were treated at a single center. A multicenter, randomized trial with a large sample size is in planning.

In conclusion, we have shown RitePrep to be superior in both bowel visualization and patient tolerability when compared to two commercially available preparations, Plenvu™ and MoviPrep®.

## Data Availability Statement

The datasets presented in this article are not readily available because individual patient data obtained as a result of this retrospective review is considered confidential and disclosure to third parties is prohibited. Data protection and data security measures were implemented for the collection, storage and processing of patient data in accordance with the principles of the GCP guidelines. Requests to access the datasets should be directed to research@cdd.com.au.

## Ethics Statement

The studies involving human participants were reviewed and approved by The ethics committee of the Centre for Digestive Diseases (approval number: CDD19/C04). The patients/participants provided their written informed consent to participate in this study.

## Author Contributions

TB, SR, and JS designed the study and oversaw the project. TB, SR, JS, and AW collected clinical data. HK-S and MD coordinated the project. HK-S collected and collated the data. HK-S and AG conducted the data analysis and drafted the manuscript. AC and AG provided support for data analysis and interpretation. All authors provided critical review of the final manuscript.

## Conflict of Interest

TB had a pecuniary interest in the Centre for Digestive Diseases. TB, SR, JS, and AW had filed patents for gastric and colonic formulations for bowel preparations. The remaining authors declare that the research was conducted in the absence of any commercial or financial relationships that could be construed as a potential conflict of interest.

## Appendix

### Suppliers

RitePrep–Sydney compounding Chemist, Australia.

Colonoscopy machine–EVIS EXERA III Xenon (CLV-190), Olympus America.

MoviPrep®–Salix Pharmaceuticals, 8540 Colonnade Center Drive, Raleigh, NC 27615, USA.

Plenvu^TM^–Norgine Limited, New Road, Hengoed, Mid Glamorgan, CF82 8SJ, United Kingdom.
