# Supplementary material for: A Comparative Clinical Study of a Novel Pre-colonoscopy Bowel Capsule Preparation Against Two Commercially Available Liquid Preparations
Source: Front Med Technol. 2021 Feb 9;2:622252. doi: 10.3389/fmedt.2020.622252 (PMC8757798; doi:10.3389/fmedt.2020.622252)
Supplement: Supplementary file 1 [file Data_Sheet_1.pdf]

<u>Instructions for Bowel Preparation Capsules</u>

These bowel preparation instructions begin the **day before procedure**

Day before procedure:

NOTHING TO <u>EAT</u> TODAY - CLEAR FLUID DIET ONLY

<u>Examples</u>

- *Water, black tea, black coffee – no Milk and no Sugar (Artificial sweeteners eg. Sugarine, equal, splenda allowed)*
- *Unsweetened 100% fruit juice – eg. Apple Juice*
- *Clear Soup – eg. Clear soup, bonox, stock cubes in water, broths, if using packet soups – strain liquid*
- *Artificially sweetened jelly – do **NOT** eat red or green coloured jelly (eg. Aeroplane jelly - jelly lite, weight watchers jelly)*

- *You may drink fizzy or bicarbonate drinks that **do not contain sugar (eg. Coke Zero, Diet Coke, Pepsi Max, mineral water)***

3 pm – Follow bowel preparation as in **<u>TABLE A - BOTTLE "A"</u>**:

Time	Bowel prep capsules	Other
**3:00pm**	6 capsules **(Bottle A)**	1 glass (250ml) of water or clear fluids
**3:30pm**	6 capsules **(Bottle A)**	1 glass (250ml) of water or clear fluids
**4:00pm**	6 capsules **(Bottle A)**	1 glass (250ml) of water or clear fluids

IF YOU OPEN YOUR BOWELS WITHIN TWO HOURS FROM THE START TIME, PLEASE FOLLOW INSTRUCTIONS IN TABLE B

<u>PLEASE DO NOT TAKE ANY MORE CAPSULES IF YOU HAVE HAD MORE THAN 15 BOWEL MOTIONS (DIARRHEA)</u>

IF YOU DO NOT OPEN YOUR BOWELS WITHIN TWO HOURS FROM THE START TIME, PLEASE FOLLOW INSTRUCTIONS IN TABLE C

<u>Please note that the bowel preparation capsules are designed to wash out the colon; symptoms such as nausea, headache, urgency and abdominal cramps may occur when you take the capsules</u>

FOR PATIENTS WHO OPEN THEIR BOWELS BY 5 PM

TABLE B - BOTTLE "B":

Time	Bowel Prep Capsules	Other
**3 hours gap**<br>1 glass (250ml) of water or clear fluids every hour		
**7:00pm**	5 capsules<br>**(Bottle B)**	1 glass (250ml) of water or clear fluids
**7:30pm**	5 capsules<br>**(Bottle B)**	1 glass (250ml) of water or clear fluids
**8:00pm**	5 capsules<br>**(Bottle B)**	1 glass (250ml) of water or clear fluids

From 8 pm you may continue drinking fluids until 12:00 am midnight.

NOTHING TO EAT OR DRINK ON THE DAY OF YOUR PROCEDURE

FOR PATIENTS WHO DO NOT OPEN THEIR BOWELS BY 5 PM

TABLE C - BOTTLES "C" & "B":

Time	Bowel Prep Capsules	Other
**1 hours gap**<br>1 glass (250ml) of water or clear fluids		
**5:00 pm**	5 capsules<br>**(Bottle C)**	1 glass (250ml) of water or clear fluids
**5:30pm**	5 capsules<br>**(Bottle C)**	1 glass (250ml) of water or clear fluids
**1.5 hour gap**<br>1 glass (250ml) of water or clear fluids		
**7:00pm**	5 capsules<br>**(Bottle B)**	1 glass (250ml) of water or clear fluids
**7:30pm**	5 capsules<br>**(Bottle B)**	1 glass (250ml) of water or clear fluids
**8:00pm**	5 capsules<br>**(Bottle B)**	1 glass (250ml) of water or clear fluids

From 8 pm you may continue drinking fluids until 12:00 am midnight.

NOTHING TO EAT OR DRINK ON THE DAY OF YOUR PROCEDURE

Please note that the bowel preparation capsules are designed to wash out the colon; symptoms such as nausea, headache, urgency and abdominal cramps may occur when you take the capsules
